# Estimation the Frequency of Human Immunodeficiency Virus among Male and Female Patients, Iran

**Published:** 2011-12-22

**Authors:** Masoud Hajia, Ali Amirzargar, Mehdi Ghoreishi, Sohrab Sam

**Affiliations:** Department of Medical Microbiology, Health Reference Laboratory of Iran, Ministry of Health and Medical Education Tehran, Iran; 2. Department of Immunology, Tehran University of Medical Sciences, Tehran, Iran; 3. Imam Khomeini Hospital, Tehran, Iran; 4. Noor Clinical Laboratory, Tehran, Iran

**Keywords:** Human Immunodeficiency Virus Frequency, Diagnosis, Medical Laboratory

## Abstract

**Objective:**

A reduction in new human immunodeficiency virus(HIV) cases is one of the ten areas prioritized by the United Nations Program on HIV. However, recent official reports confirm the HIV rate is increasing and predicted a huge incidence in the near future in Iran, despite the preventative program by Iran's Health Ministry. In this descriptive study, we evaluate the frequency of HIV positive cases among referral patients to a private clinic laboratory for its diagnosis in addition to specimens from other laboratories. An epidemiological analysis is also performed.

**Materials and Methods:**

In this descriptive study, the total number of patients was 138 cases that referred for the diagnosis of HIV to the private Laboratory. Of these, 93 males (67.4%) and 45 females (32.6%) voluntarily requested to be examined for specific increases in specific antibody titer, western blot assays and RNA quantitation polymerase chain reaction. We collected two separate tubes of whole blood, one for reverse transcriptasepolymerase chain reaction analysis and the second one for the remaining two tests. Those patients who were antibody positive by western blot and/or reverse transcriptase-polymerase chain reaction(RT-PCR) analyses were considered as HIV positive cases.

**Results:**

There were 18.84% confirmed HIVcases (17.39% males; 1.45% females). Analysis of the results confirmed that the ratio of male to female patients in the infected group was not comparable to those in the suspect group. The majority of HIV positive cases were either infected by their partner via sexual intercourse (84.61%) or needle sticks (11.53%) among the drug addicted group. The infection routes of the remainder were unknown.

**Conclusion:**

Analysis of the data revealed a higher frequency of HIVin males than females among the tested group. There was a shift in to unsafe sexual intercourse as seen in the present study. The higher rate of infected male patients shows a shift in transmission route to unsafe intercourse. Therefore, it is necessary to design new supportive programs by actively identifying and contacting at-risk groups, particularly infected females who are uninterested in being and monitored.

## introduction

Acquired immunodeficiency syndrome (AIDS) remains a leading cause of death in many countries. Injecting drug use accounts for more than two thirds (67.5%) of the reported human immunodeficiency virus (HIV) cases in Iran (1). The number of adults and children living with HIV/AIDS at the end of June 2010 was 21435 cases (92.6% male, 7.4% female), of which 46.6 % of them are of the 25 -34 year old age group (2, 3).

The spread of HIV depends on the size of the risk groups and the interaction of these groups with the general population. Evidence indicates that the number of women among people living with HIV/AIDS is lower in the Middle East and North Africa than in other regions (4). However, other recent reports show a shift in AIDS cases (5) from Africa to Asia and that it is no longer a predominantly male epidemic. Therefore, it seems the transmission route is shifting to unsafe sexual intercourse (6), resulting in an increase in its frequency among women (7-10).

Previous reports confirmed that the main route of HIV transmission in Iran was by drug use, of which about 25% had histories of imprisonment (11-13). Therefore, the Ministry of Health has taken several measures to decrease the HIV rate in this risk group, which included the implementation of a support program by the United Nations Office on Drug and Crimes (14). Despite these successful programs, recent official records confirm an increase in HIV frequency with an emphasis on the change in transmission pattern.

Iran has a young population with a rapidly increasing age at marriage that can cause an increase in HIV rate by the unsafe sexual intercourse route, although subgroups of intravenous drug users may constitute a “bridge” for transmission of HIV to the general population. Therefore, the spread of HIV depends on the size of the risk groups and the interaction of these with the general population (15).

The increasing HIV rate among women can be considered a sign for a changing HIV transmission route. In this survey, we attempt to determine the frequency of HIV and their epidemiological characteristics in referred female and male patients admitted or their specimens from other laboratories at Noor Pathobiology Laboratory.

## Materials and Methods

### Patients

This was a descriptive study performed on those patients suspected for HIV infection that they voluntarily referred for HIV diagnosis. Patients were referred by clinicians from January 2009 until December 2010. These patients consisted of people who were at risk of infection via unsafe sexual intercourse, accidental needle sticks or who were drug addicts. All patients were tested for an increase in specific HIV antibody, western blot assay, and by HIV quantitation reverse transcriptase- polymerase chain reaction (RT-PCR). In total, 138 specimens (93 male and 45 female) were received directly from referral patients or were specimens sent from other laboratories throughout Iran. Their age range at the time of receipt was 10 days to 73 years.

### Specimens

Whole blood was collected in two separate tubes (K3 EDTA; 2 ml), one for RT-PCR and the other for serology and western blot tests. Molecular examination and serology tests were examined in two different laboratories to reduce the contamination rate of the samples. All specimens were transported according to World Health Organization (WHO) recommendations (16). Molecular specimens were prepared and plasma separated in the preparation room inside the molecular laboratory, and immediately frozen at -20℃ until extraction and quantitative HIV RT-PCR. Other specimens were sent to the serology laboratory for the Enzyme- linked immunosorbent assay (ELISA) test.

### Detection of HIV antibody by ELISA

Specific HIV antibody was detected by the Genscreen Ultra HIV Ag-Ab Kit (Bio-Rad). This kit detects HIV p24 antigen and antibodies for HIV-1 and HIV-2 in human serum and plasma. The kit has been marked for in vitro diagnosis (IVD). Specificity of the kit is reported to be 99.75%, and its sensitivity on the positive sample is reported to be 100%. Detection limit of the kit is 25 pg/ml. However, every positive result was double-checked by the Western blot method as the reference method.

### Western blot assay

The MP diagnostics (MPD) HIV BLOT Kit (2.2) (MP Biomedicals Asia Pacific Pte Ltd. Cavendish Singapore) was used in this study. This kit is a qualitative enzyme immunoassay kit for the in vitro detection of antibodies to both HIV-1 and HIV-2 in human serum or plasma. The separated specific HIV-1 viral antigens incorporated onto the strips combine with a specific HIV-2 synthetic peptide. Each strip also includes an internal control as an additional control to minimize the risk of false negatives due to operational errors.

Specific antibodies to HIV-1 and HIV-2, if present in the specimens, will bind to the HIV-1 proteins and HIV-2 peptide on the strips. The strips are washed to remove the access of the antibody. Antibodies that bind specifically to HIV proteins can be visualized after removal of unbound materials by washing. This method has the sensitivity to detect marginal amounts of HIV specific antibodies in serum or plasma.

### RNA extraction

Clinical samples were extracted by the High Pure Viral RNA Nucleic Acid Purification Kit (Roche C. Mannheim, Germany).

### Quantitative **HIV RNA RT-PCR** protocols

The HIV RT-PCR Kit (DNA Technology Co., Russia) is used to detect HIV nucleic acids in two steps, the synthesis of cDNA from extracted RNA, followed by quantitative PCR test. Specific primers amplified 223 base pairs of HIV-1 genome with a specific fluorescent bicon probe labeled by fam. There is an internal control primers and probes to amplify 480 base pair of PCR product. The internal con troll probe is labeled by hex.

Extracted RNA is used immediately to synthesize cDNA by using the provided protocol from the HIV PCR Kit. Samples were incubated at 40℃ for 30 minutes, then at 95℃ for 5 minutes. In the next step, samples were centrifuged at 13000 rpm for 30 second to inactive the reverse transcriptase. A total of 5 µl of prepared cDNA was used in the quantitative PCR (Table 1). The dynamic range of the detection limit was from 300 IU-300 000 IU using the standard genome.

**Table 1:** Amplification program for **HIV PCR**


94℃	1 minute	1 cycle
94℃	20 seconds	5 cycle
58℃	15 seconds	
64℃	5 seconds	
94℃	5 seconds	40 cycle
58℃	15 seconds	
64℃	5 seconds	


Those specimens that contained HIV viral loads greater than 300,000 IU were diluted 10 fold. The 1/100 and 1/10000 dilutions were extracted to run a PCR to obtain a more accurate estimate of the viral load. In each test a low viral load of the HIV RNA (500 IU) was applied to ensure of having accurate sensitivity of the test.

### Laboratory criteria for reporting a positive **HIV** case

The following criteria were based on a recommendation from the Centre for Disease Management, Ministry of Health and Medical Education according to the following procedure (12). All specimens were firstly checked for increased specific HIV antibody. Positive specimens were tested a second time by ELISA Second, positive samples were checked by Western blot. The Western blot positive cases were checked for the presence of RNA by quantitative RT-PCR.

### Data collection analysis

All HIV-positive patients were identified by a specific code to maintain confidentiality. Patients were all interviewed for any previous HIV examinations in addition to presumed routes of infection. All analyzed data were saved on a computer spreadsheet with specific codes to ensure of safe keeping the patients records. Descriptive statistical were used for expression of data as a percentage (SPSS software Version 16).

## Results

The mean age of the suspected patients whose specimens were tested for HIV infection was 30.81 (SD±7.94), while it was 31.75 (SD±7.87) for those HIV positive patients. There were 26 patients (18.84%) identified as HIV-positive among 138 examined patients, which included 24 males (17.39%) and 2 females (1.45%).

### Gender rate among referred and infected patients

The frequency of tested specimens in the sexually active male group (20- 40 years old) was 40.56%. However, HIV-positive male patients comprised 96.12% of examined patients. Of these cases 80.76% were in the 20 -40 year old age group (Table 2). Frequency of tested female patients was 32.6%, of which only 13.76% patients were in the sexually active age group (20 -40 year old). HIVpositive female patients comprised 3.88% of cases (Table 3).

**Table 2:** Tested specimens


Age group (years)	Suspected patients (%)	
	Male	Female	Total
0-10	8.70	7.97	16.67
11-20	7.25	4.34	11.59
21-30	20.28	5.79	26.07
31-40	20.28	7.97	28.25
41-50	5.08	1.45	6.53
Over 50	7.25	3.64	10.89
Total	68.84	31.16	100


**Table 3:** Positive specimens in male and female patients


Age group (years)	Suspected patients (%)	
	Male	Female	Total
0-10	3.84	-0-	3.84
11-20	-0-	-0-	0
21-30	38.46	-0-	38.46
31-40	42.30	1.94	44.24
41-50	7.68	1.94	9.62
Over 50	3.84	-0-	3.84
Total	96.12	3.88	100


### Frequency of received specimens and positive cases from provinces

There were 86.2% of tested specimens from Tehran and the remainder (13.8%) belonged to other provinces (Table 4). The total positive cases from Tehran were 80.84%; the remaining 19.16% positive cases belonged to four provinces (Ilam, Khuzestan, Mazandaran and East Azerbaijan).

**Table 4:** Numbers of received and positive specimens from all provinces


	Province	Received specimens (%)	Positive specimens (%)
1	Alborz	1.35	-0-
2	Central	0.43	-0-
3	East Azerbaijan	2.37	4.88
4	Gholestan	1.69	-0-
5	Ilam	0.67	4.88
6	Kerman	0.43	-0-
7	Khorasan Razavi	1.69	-0-
8	Khozestan	1.69	7.32
9	Mazandaran	1.45	2.44
10	Tehran	86.2	80.48
11	Yazd	2.13	-0-
	Total	100	100


### Frequency of various transmission routes in HIV positive cases

Patients diagnosed as HIV-positive were interviewed at the laboratory to determine the transmission routes. Our records showed only 11.53% were drug addicts who were infected by needle sticks. There were 84.61% with no history of addiction, but had histories of unsafe sexual intercourse. The transmission routes of the rest were unknown.

### Frequency of received specimens and positive cases from various specialist medicines

All patients were referred by medical specialists, which included infectious diseases specialists, nephrologists, and internists (general practitioners). The frequency of identified HIV-positive cases was higher amongst patients referred by infectious disease specialists.

## Discussion

This study was based on a limited number of patients referred to a private clinical laboratory. Same studies have been previously reported on HCV frequency (17). These reports can relatively illustrate the rate of reported disease, although design a standard research is required for accurate prediction of incidence for each pathogen in community.

According to our data, HIV was confirmed in 18.84% of received specimens (1.45% female, 17.39% male). The majority of infected male patients (80.76%) were in the 20 -40 year old age range, while all infected female patients were between 31 -50 years of age (Table 3). According to the data, the ratio of suspected male to female cases was 2.14 times more, whereas the ratio of infected male to female cases was considerably more than the suspected cases.

It is reported that the frequency of HIV-positive females is 7.4% in Iran (2). This reported rate for female patients is obviously lower than the HIV-positive percentage for females worldwide (42%). Based on different Iranian published reports, HIV cases are observed mainly among drug users and prisoners, by the use of contaminated syringes and transmission to their partners via unsafe sexual contact (18, 19). In other areas of the world, HIV infection is mainly known as a sexually transmitted disease (STD) infection. Previously, a report on HIV-positive patients from a private clinic has confirmed the frequency of infected males as 82.66% and for females it was 17.34% (20), while in our study the frequency of HIV infection males was 96.12%, whereas it was 3.88% for females.

Statistical evidence indicates that the percentage of HIV-positive women is lower in the Middle East and North Africa (most under 25%) than in other regions (15). It is reported that the prevalence of HIV in this region is about 0.2%. It is lower, particularly in women, when compared with other countries. The low prevalence may be related to some cultural and religion practices that may result in differences in contact probability (i.e., the strong prohibition against extramarital sexual activities) (15). Therefore, it is suspected that those HIV-positive females who are infected have been likely infected through their marriage to a man who has engaged in risky behaviors. It is reported that the age-sex distribution of HIV confirms that women are infected at a much younger age than men in these regions. Possibly, younger women are married to older men who are more likely to have been exposed to this infection (7-9).

According to a report, voluntary testing and counseling have been established in more than 154 sites and more than 600 sites are only for voluntary counseling in Iran (18). Besides, additional efforts have had considerable impact in reducing the HIV infection rate among addicts as well as those who belong to a higher risk group when compared with the general population. Some of these efforts, such as providing free treatment for infected people, access to free syringes for addicts, and educating and encouraging the use of condoms as a physical barrier for sexually transmitted diseases.

The higher rate of referred female patients relative to male patients or the lower rate of HIV infection in women compared with men might be due to the following possibilities. Culturally, women are more sensitive to their health than men. Secondly, there is the possibility that some social prohibitions in sexual activity of extramarital partners for women are ignored, as our investigation has shown that 14 (58.33%) of the HIVpositive males were unmarried. The lower rate of addiction in women than men as well as the low rate of HIV infection in females may be underestimated because some infected patients may not be identified or monitored.

## Conclusion

The current support program of the Ministry of Health for controlling the spread of infection is believed to be successful among drug users. However, the increase in HIV-positive rate among non-addicts, particularly in males confirms a shift in HIV transmission mode and change in at-risk group, based on the results of the present study. Therefore, it is necessary to design a new supportive program by actively identifying and contacting these at-risk groups, particularly infected female patients who are not interested in being tested and monitored.
